# Overexpression of an *Agave* Phospho*enol*pyruvate Carboxylase Improves Plant Growth and Stress Tolerance

**DOI:** 10.3390/cells10030582

**Published:** 2021-03-06

**Authors:** Degao Liu, Rongbin Hu, Jin Zhang, Hao-Bo Guo, Hua Cheng, Linling Li, Anne M. Borland, Hong Qin, Jin-Gui Chen, Wellington Muchero, Gerald A. Tuskan, Xiaohan Yang

**Affiliations:** 1 Biosciences Division, Oak Ridge National Laboratory, Oak Ridge, TN 37831, USA; liudegao909@gmail.com (D.L.); rhu1@utk.edu (R.H.); zhang007jin@163.com (J.Z.); chenghua1437@126.com (H.C.); lilinling1437@126.com (L.L.); anne.borland@newcastle.ac.uk (A.M.B.); chenj@ornl.gov (J.-G.C.); mucherow@ornl.gov (W.M.); tuskanga@ornl.gov (G.A.T.); 2 The Center for Bioenergy Innovation (CBI), Oak Ridge National Laboratory, Oak Ridge, TN 37831, USA; 3 Department of Computer Science and Engineering, SimCenter, University of Tennessee Chattanooga, Chattanooga, TN 37403, USA; guohaobo@gmail.com (H.-B.G.); hong-qin@utc.edu (H.Q.); 4 School of Natural and Environmental Science, Newcastle University, Newcastle upon Tyne NE1 7RU, UK

**Keywords:** *Agave americana*, crassulacean acid metabolism, genetic engineering, *Nicotiana sylvestris*, phospho*enol*pyruvate carboxylase, photosynthesis, drought tolerance, salt tolerance

## Abstract

It has been challenging to simultaneously improve photosynthesis and stress tolerance in plants. Crassulacean acid metabolism (CAM) is a CO_2_-concentrating mechanism that facilitates plant adaptation to water-limited environments. We hypothesized that the ectopic expression of a CAM-specific phospho*enol*pyruvate carboxylase (PEPC), an enzyme that catalyzes primary CO_2_ fixation in CAM plants, would enhance both photosynthesis and abiotic stress tolerance. To test this hypothesis, we engineered a CAM-specific *PEPC* gene (named *AaPEPC1*) from *Agave americana* into tobacco. In comparison with wild-type and empty vector controls, transgenic tobacco plants constitutively expressing *AaPEPC1* showed a higher photosynthetic rate and biomass production under normal conditions, along with significant carbon metabolism changes in malate accumulation, the carbon isotope ratio δ^13^C, and the expression of multiple orthologs of CAM-related genes. Furthermore, *AaPEPC1* overexpression enhanced proline biosynthesis, and improved salt and drought tolerance in the transgenic plants. Under salt and drought stress conditions, the dry weight of transgenic tobacco plants overexpressing *AaPEPC1* was increased by up to 81.8% and 37.2%, respectively, in comparison with wild-type plants. Our findings open a new door to the simultaneous improvement of photosynthesis and stress tolerance in plants.

## 1. Introduction

Human population increases, global climate changes and natural resources reductions could seriously impact food and energy security in the future [1,2,3,4,5,6,7,8]. The drylands, with precipitation amounts that are inadequate for most present-day food and bioenergy crops, covers approximately 40% of the world’s land [9,10]. Moreover, around 20% of irrigated areas in the world are under salt stress which has become a big constraint limiting agricultural production [11,12,13]. In addition, the phytotoxic effects of nanoparticles could impact plant growth [14,15]. To address these challenges, tremendous efforts have been put into improving photosynthesis, drought tolerance and salt tolerance in crop plants through breeding and genetic engineering over the past 50 years, though there has been limited success in simultaneously enhancing both biomass production and stress tolerance [16]. One common problem shared by previous breeding and genetic engineering approaches is that they have focused on drought- or salt-responsive genes in C_3_ or C_4_ species, with narrow genetic diversity for the tolerance of abiotic stresses.

A potential solution to this problem can be obtained from the desert plants that employ crassulacean acid metabolism (CAM) for photosynthesis (e.g., *Agave americana*), as these plants maximize water-use efficiency (WUE) by shifting all or part of their net atmospheric carbon dioxide (CO_2_) uptake to the nighttime, when the evapotranspiration rates are generally lower than that in the daytime [1,2,8,9,17]. The nocturnal CO_2_ taken up in CAM plants is intracellularly fixed, usually leading to the generation of C_4_ organic acid (e.g., malate) as storage intermediates, which accumulate in the vacuole during the dark period before being gradually catabolized during the day [2,9]. High rates of malate decarboxylation behind closed stomata during the day can concentrate CO_2_ from 2- to 60-fold around ribulose-1–5-bisphosphate carboxylase/oxygenase (Rubisco) [9,18]. This effectively creates a ‘CO_2_ pump’ that can strongly favor the carboxylase activity of Rubisco and potentially suppress photorespiration, a process that can reduce photosynthesis by up to 40% in C_3_ photosynthesis plants [1,2,9,19,20,21]. In addition, as an obligate CAM species, *Agave americana* is highly tolerant to salinity with an electrical conductivity (EC) level of 9.4 dS m^−1^ [22]. Additionally, *A. americana* has been reported to achieve high biomass productivity and drought tolerance when grown as a crop on arid and semi-arid lands [23].

CAM photosynthesis can be divided into nocturnal and day-time reactions [24,25,26,27]: At night atmospheric CO_2_ is taken up through open stomata and primary fixation of CO_2_ (as HCO_3_^−^) by phospho*enol*pyruvate carboxylase (PEPC) produces oxaloacetate (OAA) that is subsequently converted to malate by malate dehydrogenase (MDH). Malate is transported by tonoplast-localized aluminum-activated malate transporter (ALMT) into the vacuole, where it is stored as malic acid throughout the dark period. During the light period, Rubisco fixes the CO_2_ released from NAD(P)-malic enzyme (ME)- or PEP carboxykinase (PEPCK)-mediated malate decarboxylation when stomata are closed [9,19]. It has been shown that PEPC is a highly abundant enzyme in leaves of *A. americana* and its transcript level and protein abundance are correlated with CAM [28]. In this study, we identified a CAM-isoform *PEPC* through genome-wide analysis of the *PEPC* gene family in *A. americana,* as well as protein structure modeling analysis and molecular dynamics (MD) simulation studies. We then determined the impacts of the overexpression of the *Agave PEPC* on the photosynthetic carbon metabolism, and abiotic stress tolerance in the model C_3_ plant tobacco.

## 2. Materials and Methods

### 2.1. Genome-Wide Analysis of the PEPC Gene Family

*Arabidopsis thaliana PEPC* sequences (AT1G68750.1, AT1G53310.1, AT3G14940.1, and AT2G42600.2) were retrieved and used as queries in BLAST searches against *Agave americana* transcriptomics data [28] to identify potential *PEPC* genes. An expectation (E) value of <1 × 10^−10^ was used to obtain the homologous protein sequences of the predicted *PEPC* family members. The expression data of the *Agave* PEPCs were also obtained from the *A. americana* transcriptomics data [28]. For gene expression patterns of *Agave PEPC*, the log10 transformed FPKM values and z-score normalized relative expression were used for heatmap analysis.

### 2.2. Phylogenetic Analysis

Multiple alignment of PEPC proteins was performed using the MAFFT online service [29]. The maximum likelihood (ML) phylogenetic tree was constructed using W-IQ-TREE [30]. The sequences used for the analysis were: foxtail millet (AY491400), foxtail millet C_4_ (AF495586), maize C_3_ (X61489), maize C_4_ (X15642), maize root (AB012228), rice C_3_ (Os08g0366000, Os09g0315700, and Os01g0758300), rice root (Os02g0244700), sorghum C_4_ (X63756), sugarcane C_3_ (M86661), sugarcane C_4_ (AY135709), and wheat (AJ007705) [31]. The bootstrap values were calculated as percentages for 1000 replications.

### 2.3. Structural Modeling and Molecular Dynamics Simulation

The *A. americana* PEPC model was constructed using the I-TASSER v. 5.1 protein structure modeling toolkit [32]. Structure averaging from multiple MD simulations or a single long time-scale MD simulation could effectively refine the predicted structures [33]. Here, starting with the best I-TASSER model, the rotamer states of Asn, Gln, and His residues as well as protonation states of titratable residues were validated by MolProbity [34]. The HBUILD module in CHARMM [35] was employed to add missing hydrogen atoms to the model. A water box with the size of 118 × 106 × 102 Å3 (at least 15 Å to the edge of the protein) was used, and sodium and chloride ions were added to neutralize the net charge. The final model contained 120,210 atoms with 34,893 water molecules. NAMD [36] was used to perform the MD simulations with the CHARMM protein force field [37] and TIP3P water model [38]. Using the SHAKE algorithm to fix all bond lengths involving hydrogen atoms, a time step of 2-fs was applied to all MD simulations. A 50k-step energy minimization was conducted, followed by a “natural” heating to 300 K with the rate of 0.001 K/step for 300 k steps. An NPT ensemble maintained by Langevin piston controls was used in the MD simulations with a system pressure of 1 atm and a temperature of 300 K. A cutoff switching between 9 and 11 Å was applied for non-bonded interactions. The particle mesh Ewald (PME) summation was applied for long-range electrostatic interactions with a grid spacing of 1.35 Å. MD simulation was performed for 200 ns, and analysis was carried out on the final 50 ns of the MD trajectory. The PDBsum online tool [39] was used to plot the cartoon topology of the protein structure.

### 2.4. Plasmid Construction

A 2943-bp DNA fragment containing the coding sequence of *AaPEPC1* (Aam080248) [28] fused to two FLAG epitope tags [40] was chemically synthesized by Integrated DNA Technology (Coralville, IA, USA) and used to produce a chimeric gene construct, p35S:*FLAG-AaPEPC1*/pNOS: *nptII*. The vector contains the CaMV35S promoter which drives *FLAG-AaPEPC1,* and the nopaline synthase (NOS) promoter which drives the *nptII* gene for kanamycin resistance as a selection marker. The vector was delivered into the GV3101 *Agrobacterium tumefaciens* strain via the freeze-thaw method [41] for plant transformation.

### 2.5. Plant Transformation

A tobacco (*Nicotiana sylvestris*) cultivar (USNGC TW136, PI555569) was used for genetic transformation. The generation and culturing of transgenic plants were carried out as previously described [42]. The transgenic lines were based on single copy lines with a segregation ratio of approximately 3:1 (kanamycin resistance vs. sensitivity) in the T1 generation, and homozygous lines were presumed if there was no segregation in the T2 and T3 generations (n > 100).

### 2.6. Measurement of Photosynthesis

The photosynthetic rates of leaves of transgenic and wild-type (WT) plants that were well-watered and grown in pots for 6 weeks was measured as previously described [11]. Mature leaves from 3 individual replicate plants were used for gas exchange analysis by an LI-COR Portable Photosynthesis System (LI-COR Inc., Lincoln, NE, USA). The relative chlorophyll content (SPAD value in fresh leaves) was analyzed using an SPAD-502 Chlorophyll Meter (Minolta, Japan) [11].

### 2.7. Analysis of Malate, Glucose and Proline Content

Mature leaves were sampled and frozen in liquid nitrogen at the indicated times and stored at −80 °C until use. The leaf samples were ground to a fine powder and assayed for malate and glucose content using the standard enzyme-linked spectrophotometric methods according to the manufacturer’s instructions (Sigma-Aldrich, St. Louis, MO, USA), respectively.

The proline content was measured as described by He, et al. [43].

### 2.8. Carbon Isotope Ratio Analysis

Plants were well watered throughout the growing period. Mature leaves were harvested from 6-week-old plants and dried for 1 week at 50 °C. Finely ground dry powder was placed in capsules and then analyzed at the University of California Davis Stable Isotope Facility (http://stableisotopefacility.ucdavis.edu, accessed on 25 February). 

### 2.9. Salt and Drought Stress Treatment

For salt tolerance analysis, the transgenic plants and controls were watered with 200 mM NaCl solution every other day for 4 weeks as previously described [11]. For drought tolerance analysis, the transgenic and WT plants were exposed to progressive drought stress by withholding water until a nearly lethal effect of dehydration was observed on the WT. A recovery study was carried out for plants under drought stress by re-irrigating with water [44]. After salt or drought treatment, all plants were dried for 48 h in an oven at 80 °C and weighed [11]. All treatments were performed in triplicate.

### 2.10. Expression Levels Analysis of the Related Genes

The expression levels of related genes in the transgenic and WT plants were analyzed by qRT-PCR as previously described [11]. Total leaf RNA was extracted from transgenic and WT plants using the Spectrum Plant Total RNA Kit (Sigma-Aldrich, St. Louis, MO, USA). RNA samples were reverse-transcribed using a High-Capacity cDNA Reverse Transcription Kit (Applied Biosystems, Foster City, CA, USA). The cDNA solution was used as a template for qPCR amplification using SYBR Green Master Mix (Applied Biosystems, Foster City, CA, USA) with the specific primers designed for each gene (Supplementary Table S1). The tobacco *actin* gene was used as an internal control (Supplementary Table S1). The quantification of the gene expression level was performed with comparative CT method [45].

### 2.11. Statistical Analysis

The data presented as the mean ± SD were analyzed by a one-way ANOVA analysis with post-hoc Tukey honestly significant difference (HSD) test. *p-*values of <0.05 or <0.01 were considered to be statistically significant.

## 3. Results

### 3.1. CAM-Specific PEPC in Agave americana

**Figure 1:**
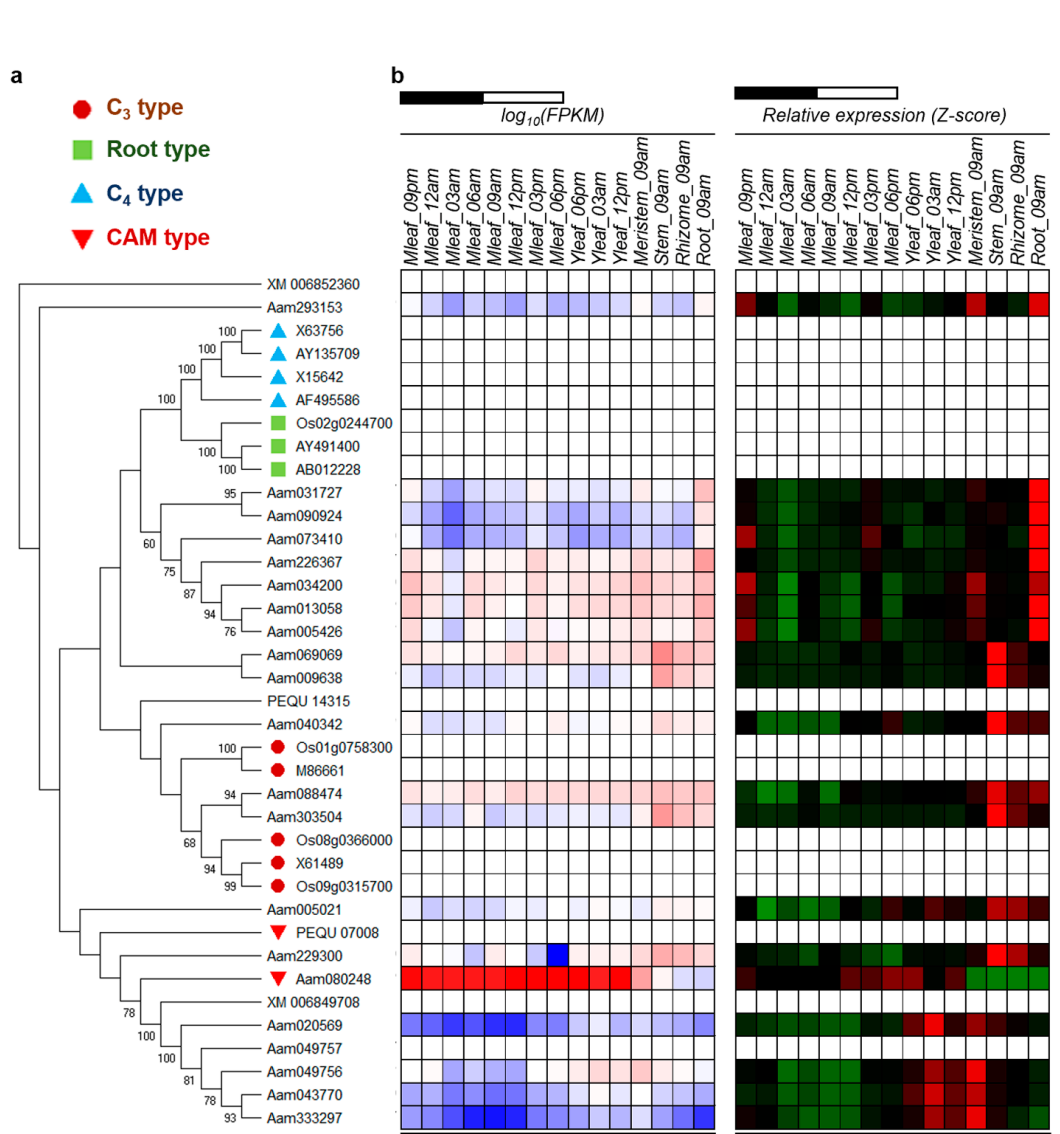
Identification of the crassulacean acid metabolism (CAM)-isoform of phospho*enol*pyruvate carboxylases (PEPC) in *Agave americana. (***a**) Phylogenetic relationships of PEPCs in *A. americana* and plant-type PEPCs from other monocot plants. Bootstrap values are shown at nodes. The sources for the PEPC sequences (as well as species abbreviations, etc.) are provided in ‘Materials and Methods’. (**b**) Diel transcript expression of the *PEPC* genes in *A. americana*. The Z-score is defined as (x_i_ − μ_i_)/σ_i_, where x_i_ is the FPKM of gene i, μ_i_ is the mean and σ_i_ is the standard deviation of all 15 columns. White and black bars indicate daytime (12 h) and nighttime (12 h), respectively. Mleaf: mature leaf; Yleaf: young leaf.

Through a tBLASTn search against *A. americana* transcriptomics data [28], using the PEPC protein sequences of *Arabidopsis thaliana* as queries, we identified a total of 21 transcripts encoding PEPC in *A. americana*. Several types of PEPC are present in plants, including plant-type PEPCs (PTPCs) and one bacterial-type PEPC (BTPC) [46,47]. The plant-type PEPCs studied so far have been classified into four groups: C_3_, C_4_, and CAM-types from photosynthetic tissues and root-type from non-photosynthetic tissue [31]. In order to gain insight into the evolutionary relationships among PTPCs, we constructed a phylogenetic tree using the 21 predicted *Agave PEPC* transcripts and the C_3_-, C_4_-, CAM- and root-type PEPCs from rice (*Oryza sativa*), maize (*Zea mays*), wheat (*Triticum aestivum*), sugarcane (*Saccharum* spp.), sorghum (*Sorghum bicolor*), foxtail millet (*Setaria italica*) and orchid (*Phalaenopsis equestris*) [24,31]. A phylogenetic analysis of the plant-type PEPCs indicated that Aam080248 [28] (named AaPEPC1) belongs to CAM-type PEPC (Figure 1a). Our results also showed the CAM- and C_4_-type PEPCs belong to different clades, suggesting that these two PEPC types evolved independently.

Using the quantitative gene transcript abundance data obtained from the transcriptome analysis of 15 tissues, including mature leaves sampled over a diel cycle (eight time-points) and, young leaves (three time-points), meristem, stem, root and rhizome in *A. americana* [28], we obtained the transcript pattern of the 21 *PEPC* transcripts (Figure 1b), Among which Aam080248 was the most abundant transcript in mature leaves, with a transcript abundance peak during the late afternoon just before the start of the dark period. These results supported the above computational phylogenetic prediction (Figure 1a) that the Aam080248 gene encodes the CAM-specific PEPC in *A. americana*.

### 3.2. AaPEPC1 Binds to Phosphoenolpyruvate

**Figure 2:**
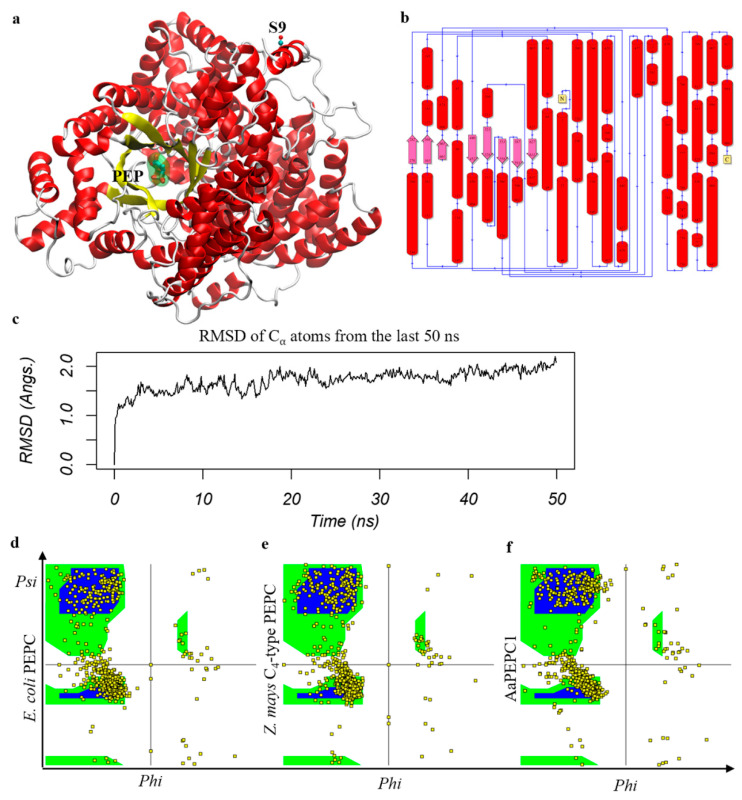
AaPEPC1 binds to phospho*enol*pyruvate (PEP) substrate. (**a**) Left: AaPEPC1 structure selected from 200 ns molecular dynamics (MD) simulation that is closest to the average structure of the whole trajectory (root mean square deviation RMSD = 0.8 Å). The protein structure is shown in cartoons with helices in red, strands in yellow and coils/turns in white. A PEP substrate that binds to the β-barrel (yellow) active site is shown in sticks and spheres. The plant-specific N-terminal serine residue (S9) is also shown. The binding position of the PEP substrate was obtained from the *Escherichia coli* PEPC–PEP complex (PDB entry 1JQN) [50]. (**b**) A cartoon representation of the topology of AaPEPC1 structure. The cylinders represent alpha-helices. (**c**) The RMSD (Å) profile of the final 50 ns of the MD simulation. The deviations of all snapshots to the average MD structure are 1.4 ± 0.2 Å (not shown). (**d**–**f**) Ramachandran plot of the *E. coli* (PDB entry 1JQN) PEPC, *Zea mays* C_4_-type PEPC (PDB entry 5VYJ) [49], and AaPEPC1 (the final snapshot of the 200 ns MD trajectory), respectively.

Phospho*enol*pyruvate (PEP) is the substrate for PEPC enzymes [48,49]. To understand whether AaPEPC1 binds to PEP, we developed a protein structural model using I-TASSER (v.5.1) accompanied by a 200 ns MD simulation (Figure 2a–c). The last 50 ns of the MD trajectory were taken to refine the AaPEPC1 structure. The root mean square deviation (RMSD) of the α-carbon atoms of all residues was approximately 2.0 Å (Figure 2c), which is reasonable for fluctuations of the 964 amino acids (AA) protein. From the last 50 ns MD trajectory, the snapshot that was closest to the average structure (with RMSD = 0.8 Å) was selected as the final model (Figure 2a,b). Since the crystal structures of bacterial (*Escherichia coli*) PEPC (PDB entry 1JQN) and *Z. mays* C_4_-type PEPC (PDB entry 5VYJ) are well-characterized [48,49,50], we compared the protein structure of AaPEPC1 with the *E. coli* PEPC and *Z. mays* C_4_-type PEPC. In line with *E. coli* PEPC and *Z. mays* C_4_-type PEPC, the AaPEPC1 model was found to be dominated by α-helix regions (546 AAs, 57.7%). The Ramachandran plot of the AaPEPC1 structure was found to have 93.4% (804 AAs) in the most favorable regions, compared to 92.6% for the *E. coli* PEPC and 90.5% for the *Z. mays* C_4_-type PEPC (Figure 2d–f). In addition, 5.2% (45 AAs) of AaPEPC1 were found to be in the additional allowed regions (7.1% for *E. coli* PEPC and 8.7% for *Z. mays* C_4_-type PEPC), 1.2% (10 AAs) were found to be in the generously allowed regions (0.1% for *E. coli* PEPC and 0.2% for *Z. mays* C_4_-type PEPC) and 0.2% (2 AAs) were found to be in disallowed regions (0.1% for *E. coli* PEPC and 0.3% for *Z. mays* C_4_-type PEPC). The N-terminus of AaPEPC1 was found to contain the plant-specific serine residue (S9) (Figure 2a), which is located in the middle of an α-helix flanked by a long loop region. The plant-specific Ser residue near the N-terminus of PEPC is well-known in C_4_ plant species [48,51]. This serine residue, however, was absent in bacterial PEPC (e.g., *E. coli* PEPC, PDB entry 1JQN) (Figure 2a,b). The AaPEPC1 model suggested that the AaPEPC1 can efficiently bind to PEP, which is bound by H171 and R640, where R640 is located at a GRGGXXGR^640^GG motif (Figure 2a) and is overlapped with R647 in the *Z. mays* C_4_-type PEPC, whereas H171 is overlapped with H177 in the *Z. mays* C_4_-type PEPC, hence both H171 and R640 in AaPEPC1 may directly participate in the carboxylation reaction as proposed in the C_4_-type PEPC [48]. In addition, based on the PEP-AaPEPC1 complex model, R449 (R456 in *Z. mays* C_4_-type PEPC) may also be involved in PEP binding and PEPC catalysis.

### 3.3. Development of Transgenic Tobacco Lines Overexpressing AaPEPC1

After we identified the CAM-isoform of *PEPC* from *A. americana*, we wanted to determine the impact of overexpressing this *Agave* gene on the photosynthetic carbon metabolism and abiotic stress tolerance in *Nicotiana sylvestris* (tobacco), which is a C_3_ plant species. The *AaPEPC1* coding sequence was cloned into binary vector pBI121, downstream of the cauliflower mosaic virus 35S (CaMV35S) promoter, to yield p35S::AaPEPC1 for transformation into *N. sylvestris* (Supplementary Figure S1a). The p35S::AaPEPC1 vector and empty vector (pBI121) were transferred into tobacco via *A. tumefaciens*-mediated genetic transformation [42]. Transformants harboring a single copy of transgene were identified from the segregation ratio for kanamycin resistance. Two T2 transgenic homozygous lines (p35S::AaPEPC1_OE1 and p35S::AaPEPC1_OE2) exhibiting different expression levels of the *AaPEPC1* (Supplementary Figure S1b) were selected as representative lines for subsequent phenotypic characterization.

### 3.4. Overexpression of AaPEPC1 Increases Photosynthetic Rate and Changes Stable Carbon Isotope Ratio

**Figure 3:**
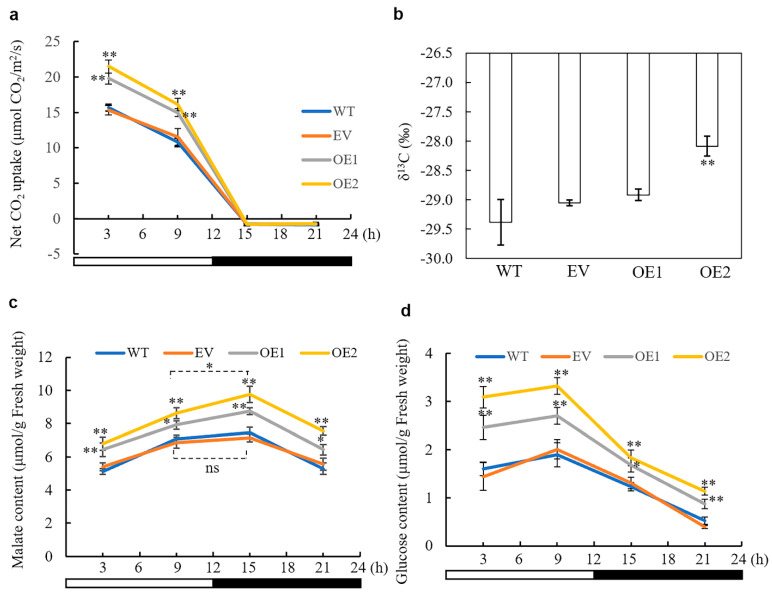
Impact of overexpressing *AaPEPC1* on photosynthetic rate (**a**), carbon isotope ratio δ^13^C (**b**), malate content (**c**), and glucose content (**d**) in tobacco. OE1 and OE2: the transgenic plants expressing *AaPEPC1.* EV: empty vector control. WT: wild-type plants. White and black bars indicate the light period (12 h) and the dark period (12 h), respectively. *X*-axis represents the time after the beginning of the light period at 00:00 h. Values represent means ± SD (n = 3 individual replicate plants). * and ** indicated significant difference from that of WT at *p* < 0.05 and *p* < 0.01, respectively, by one-way ANOVA analysis with a post-hoc Tukey honestly significant difference (HSD) test. Ns = non-significant.

We determined whether the constitutive overexpression of *AaPEPC1* affected or influenced the photosynthetic rate in the transgenic tobacco plants, along with empty vector (EV) and WT control plants, grown under normal conditions (12 h light/12 h dark photoperiod; without drought- or salt-stress) at four time points (i.e., 3, 9, 15 and 21 h after the beginning of the light period). The transgenic plants overexpressing *AaPEPC1* showed significantly higher photosynthetic rates than the WT and EV controls in the light period (Figure 3a). A carbon isotope ratio δ^13^C is a broadly accepted indicator of the extent to which the biomass is derived from PEPC-mediated CO_2_ fixation in plants, because PEPC discriminates less against ^13^C than Rubisco which is responsible for most CO_2_ fixation in C_3_ plants during the light period [52]. The positive correlation between the δ^13^C values and CAM activity has been demonstrated to be a simple and reliable method for determining the type of photosynthesis, including that of C_3_, C_3_-CAM intermediate and CAM [52,53,54]. In this study, we found that the δ^13^C values were significantly increased (i.e., became less negative) in the transgenic line (OE2), in which the expression level of *AaPEPC1* was 7.74-fold higher than that in the other transgenic line (OE1) (Supplementary Figure 1b), in comparison with the controls (Figure 3b), thus indicating that this transgenic line was using PEPC for photosynthetic carbon assimilation and production of biomass.

### 3.5. The Impact of AaPEPC1 Overexpression on the Accumulation of Malate and Glucose

PEPC functions in the production of OAA, thus leading to synthesis of malate/malic acid, which is a key intermediate of the tricarboxylic acid (TCA) cycle that links lipids and glucose metabolisms with photosynthesis in C_3_ plants [47,55]. The diel fluctuation in malate content represents a central biochemical correlate of the CAM photosynthesis pathway [9]. Additionally, the glycolytic breakdown of glucose provides substrate for the primary carboxylation reaction at night whilst the day-time recovery of glucose via gluconeogenesis is an important sink for C released from malate decarboxylation [9,56]. To determine the impact of *AaPEPC1* overexpression on malate and glucose production in transgenic tobacco, the malate and glucose contents were measured at four time points (i.e., 3, 9, 15 and 21 h after the beginning of the light period). The transgenic plants overexpressing *AaPEPC1* showed higher malate and glucose contents than the WT and EV controls at all four time points during the day and night (Figure 3c,d). Furthermore, the transgenic plants expressing *AaPEPC1* showed a significant increase in malate content at 15 h compared to that at 9 h after the beginning of the light period, while no significant difference was found between the malate contents at 15 and 9 h in the control plants (Figure 3c). The rewired diel malate accumulation–depletion pattern suggested that *AaPEPC1* overexpression affects primary carboxylation in transgenic plants. Additionally, we found that higher amounts of glucose were broken down in the early night (at 15 h after the beginning of the dark period) in the transgenic plants overexpressing *AaPEPC1* compared with control plants (Figure 3d), suggesting that the overexpression of the *AaPEPC1* enhances glycolysis and consequently supplies more substrates for primary carboxylation.

### 3.6. CAM-Related Genes Were Up-Regulated by AaPEPC1 Overexpression

**Figure 4:**
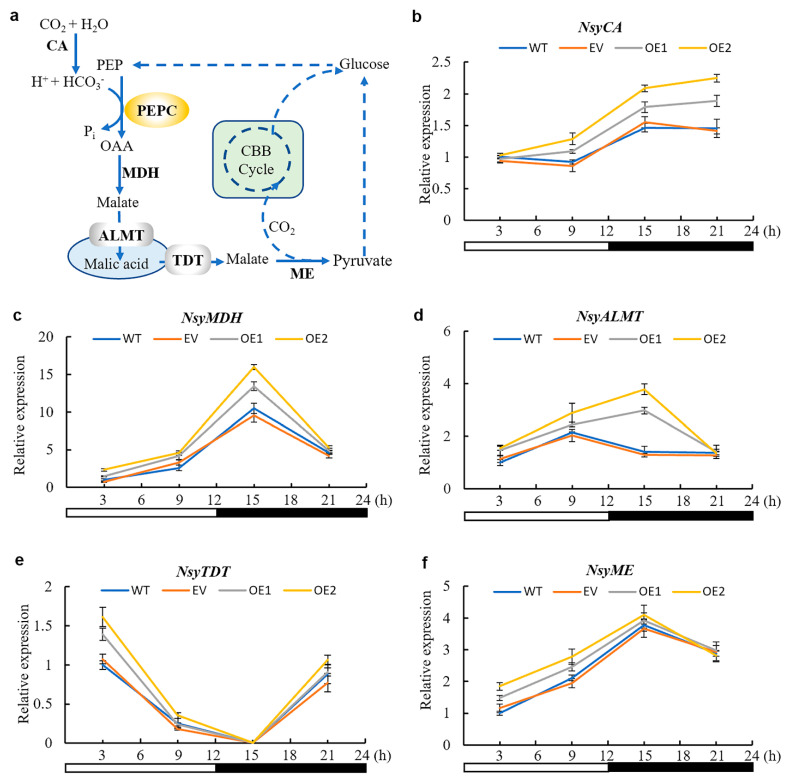
Relative expression level of the orthologs of CAM pathway genes in *AaPEPC1*-overexpressing tobacco plants. (**a**) Diagram of the CAM pathway in malic enzyme (ME) subtype [24]. CA: carbonic anhydrase; PEPC: phospho*enol*pyruvate carboxylase; OAA: oxaloacetate; MDH: malate dehydrogenase; ALMT: aluminum-activated malate transporter; TDT: tonoplast dicarboxylate transporter; CBB: Calvin-Benson-Bassham. (**b**–**f)** Relative expression level of *Nicotiana sylvestris* CA (*NsyCA*, XM_009805732.1), *NsyMDH* (XM_009784202.1), *NsyALMT* (XM_009797046.1), *NsyTDT*, (XM_009797970.1) and *NsyME* (XM_009781546.1), respectively. The *actin* gene (XM_009774717.1) was used as an internal control. The values were normalized to expression in the wild-type plants (WT) at the 3 h light time-point. White and black bars indicate daytime (12 h) and nighttime (12 h), respectively. *X*-axis represents the time after the beginning of the light period. Values represent means ± SD (n = 3).

To test whether the re-programed changes in diel malate and glucose content led to feedback regulation of CAM pathway genes, the transcript abundance of the orthologs of CAM genes in the transgenic and WT plants was analyzed using qRT-PCR (Figure 4a). The expression of carbonic anhydrase (NsyCA), an ortholog of CA that is responsible for rapid interconversion of CO_2_ and HCO_3_^−^ in CAM, was more increased in transgenic plants expressing *AaPEPC1* compared with that in the controls at 9, 15 and 21 h after the beginning of the light period (Figure 4b). The expression levels of malate dehydrogenase (*NsyMDH*) (an ortholog of MDH that is responsible for catalyzing the oxidation of malate to OAA in CAM) and tonoplast aluminum-activated malate transporter (*NsyALMT*) (an ortholog of ALMT that is responsible for transporting malate into vacuole in CAM) in the transgenic plants expressing *AaPEPC1* were higher than those in the controls at the early night period (15 h after the beginning of the light period)(Figure 4c,d). The expression of tonoplast dicarboxylate transporter (*NsyTDT*) (an ortholog of TDT that may be responsible for transporting malic acid out of vacuole in CAM) was up-regulated in transgenic plants expressing *AaPEPC1* at the early morning period (3 h after the beginning of the light period) (Figure 4e). The expression level of malic enzyme (*NsyME*) (an ortholog of ME that may be responsible for the decarboxylation of malate in CAM species) in transgenic *AaPEPC1* plants was increased during the light period (Figure 4f). These results demonstrated that the overexpression of the *AaPEPC1* up-regulates the expression of the orthologs of CAM pathway genes in transgenic plants.

### 3.7. Impact of AaPEPC1 Overexpression on Biomass Production

**Figure 5:**
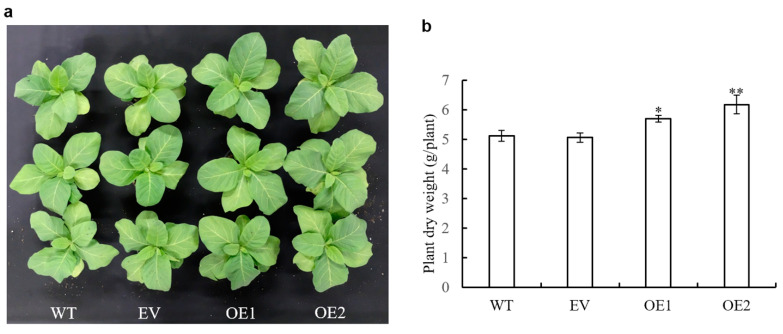
Growth characteristics of transgenic tobacco plants expressing *AaPEPC1*. (**a**) Phenotypes of transgenic plants expressing *AaPEPC1* (OE1 and OE2) as well as empty vector (EV), and wild-type plants (WT), grown in pots for 6 weeks under well-watered conditions. (**b**) Dry weight (shoot and root) of transgenic plants and WT. Values represent means ± SD (n = 3). * and ** indicate significant difference from that of WT at *p* < 0.05 and *p* < 0.01, respectively, by one-way ANOVA analysis with post-hoc Tukey HSD.

Based on the above, we speculated that the higher photosynthetic rates, glucose content and carbon isotope ratio δ^13^C measured in transgenic plants overexpressing *AaPEPC1* may result in improved biomass production. To test this, we examined the growth of the transgenic plants overexpressing *AaPEPC1*, along with the EV and WT control plants, in growth chambers under well-water conditions. After six weeks of growth, the transgenic *AaPEPC1* plants exhibited larger physical size than the WT and EV controls (Figure 5a). The dry weight of the transgenic *AaPEPC1* plants was significantly increased in comparison with the control plants (Figure 5b), demonstrating that *AaPEPC1* overexpression increases biomass production in transgenic plants under normal conditions.

### 3.8. Impact of AaPEPC1 Overexpression on Salt and Drought Tolerance

**Figure 6:**
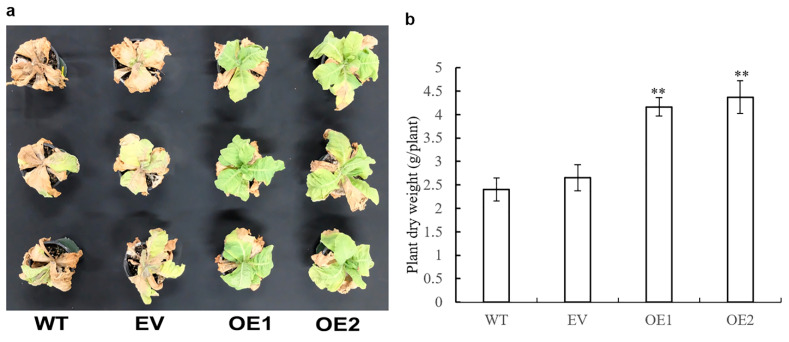
Responses of the *AaPEPC1*-overexpressing tobacco plants under salt stress. (**a**) Phenotypes of transgenic plants expressing *AaPEPC1* (OE1, OE2) or empty vector (EV), and wild-type plants (WT) grown in pots under 200 mM NaCl stress. The plants were irrigated with 200 mM NaCl solution once every 2 days for 4 weeks. (**b**) Dry weight of transgenic plants and WT. Values represent means ± SD (n = 3). ** significant difference from that of WT *p* < 0.01, by one-way ANOVA analysis with post-hoc Tukey HSD.

Most crop plants are susceptible to salinity. The NaCl stress at concentrations of 100–200 mM can inhibit or even completely prevent plant growth, resulting in their death [57,58]. To investigate whether the overexpression of *AaPEPC1* enhanced salt tolerance in transgenic plants, the transgenic *AaPEPC1* plants as well as the EV and WT controls were grown in pots and irrigated with 200 mL of 200 mM NaCl solution once every two days for four weeks. The salt-stress treatment caused the death of the WT and EV control plants, while the transgenic plants overexpressing *AaPEPC1* maintained growth (Figure 6a). The dry weight of the transgenic plants overexpressing *AaPEPC1* was significantly increased compared to the controls (Figure 6b), establishing that the overexpression of *AaPEPC1* significantly enhanced tolerance to salt stress in transgenic tobacco plants.

**Figure 7:**
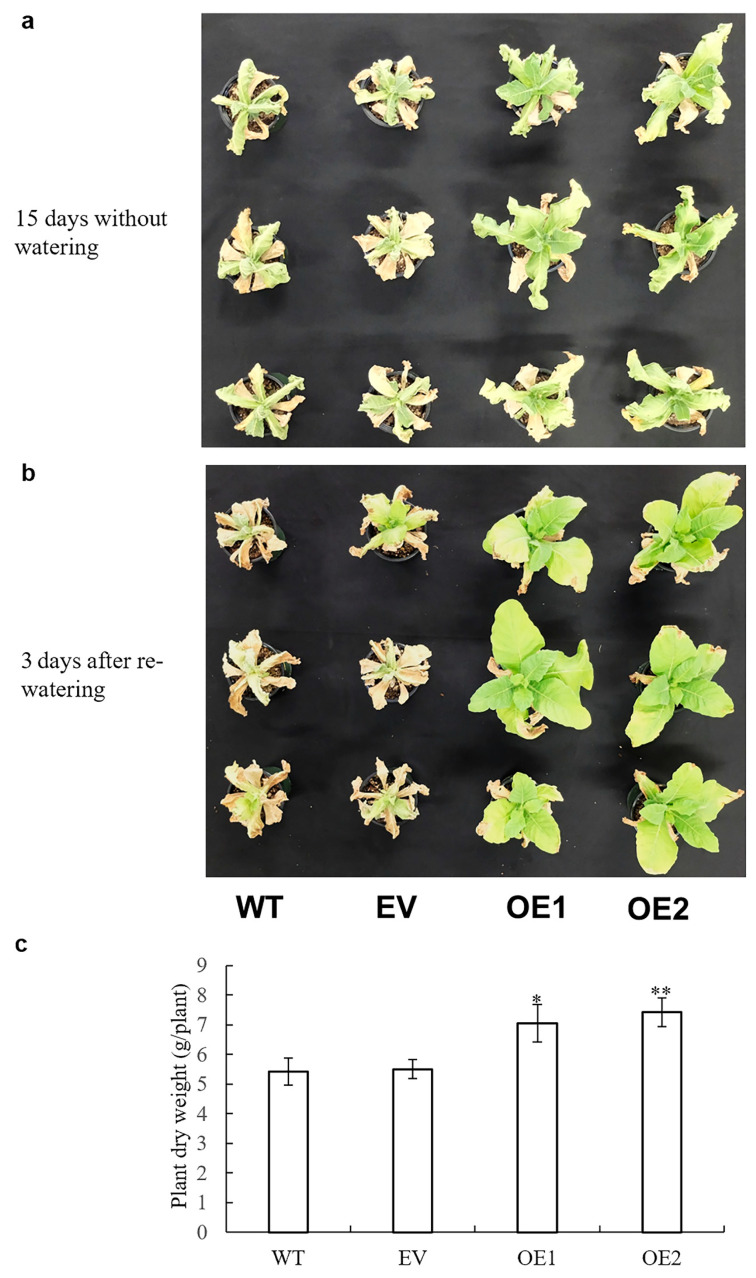
Responses of the *AaPEPC1*-overexpressing tobacco plants under drought stress. Phenotypes of transgenic plants expressing *AaPEPC1* (OE1 and OE2) or as well as empty vector (EV), and wild-type plants (WT) grown in pots under drought stress (**a**) and after re-watering (**b**). Transgenic and WT were grown in soil for 15 days without watering. (**c**) Dry weight of transgenic and WT plants. Values represent means ± SD (n = 3). * and ** indicate significant difference from that of WT at *p* < 0.05 and *p* < 0.01, respectively, by one-way ANOVA analysis with post-hoc Tukey HSD.

To investigate whether overexpression of *AaPEPC1* enhanced drought tolerance in transgenic plants, the transgenic *AaPEPC1* plants, and EV and WT controls, were subjected to drought stress in growth chamber condition. After 15 days without watering, all WT and EV control plants displayed severe wilting (all leaves were severely curled and most leaves had turned yellow/or were dead), whereas the growth of transgenic tobacco plants expressing *AaPEPC1* was less affected and their youngest leaves were still green and expanded (Figure 7a). Three days after re-watering, all WT and EV controls were nearly dead, whereas all transgenic lines expressing *AaPEPC1* survived and started to regrow (Figure 7b). Dry weight of the transgenic plants expressing *AaPEPC1* was also significantly increased compared to the WT and EV controls (Figure 7c), providing evidence that overexpression of *AaPEPC1* improved the drought tolerance in the transgenic tobacco plants.

### 3.9. Proline Biosynthesis Is Enhanced by AaPEPC1 Overexpression

**Figure 8:**
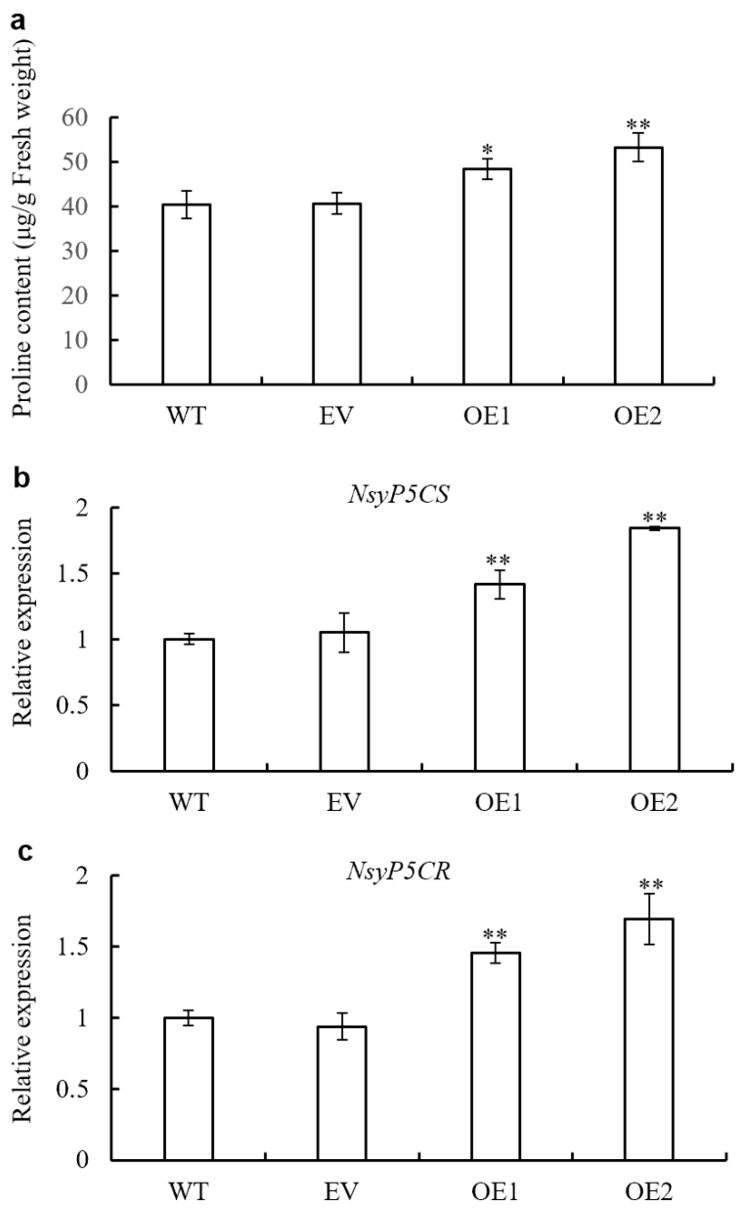
Changes in proline biosynthesis in *AaPEPC1*-overexpressing tobacco plants. (**a**) Proline content in the leaves of transgenic plants expressing *AaPEPC1* (OE1 and OE2) as well as empty vector (EV), and wild-type plants (WT). (**b**) and (**c**) Relative expression level of the proline biosynthesis genes pyrroline-5-carboxylate synthase (P5CS) and pyrroline-5-carboxylate reductase (P5CR) in the *AaPEPC1*-overexpressing tobacco plants. Values represent means ± SD (n = 3). * and ** indicate significant difference from that of WT at *p* < 0.05 and *p* < 0.01, respectively, by one-way ANOVA analysis with post-hoc Tukey HSD.

PEPC plays a crucial role in nitrogen metabolism in *Arabidopsis* [47] and loss-of-function of both *PEPC1* and *PEPC2* decreased the levels of glutamate in *Arabidopsis*. Glutamate can be converted into proline through pyrroline-5-carboxylate synthase (P5CS) and pyrroline-5-carboxylate reductase (P5CR) [11]. Proline plays important roles in stress tolerance, e.g., drought and salt stress tolerance, by regulating osmotic balance, activating the ROS scavenging system, protecting membrane integrity and photosynthesis [11,59]. We hypothesized that overexpression of *AaPEPC1* could enhance proline biosynthesis, and consequently increase the drought and salt stress tolerance in the transgenic plants. To test this hypothesis, proline content was analyzed in the transgenic *AaPEPC1* plants, along with the WT and EV controls. The proline contents in the transgenic plants expressing *AaPEPC1* were significantly higher than that in the WT and EV controls (Figure 8a). Furthermore, the expression levels of two key proline biosynthesis genes *NsyP5CS* and *NsyP5CR* were significantly higher in the transgenic *AaPEPC1* plants in comparison with the WT and EV controls (Figure 8b,c), supporting our hypothesis that the overexpression of *AaPEPC1* increased drought and salt stress tolerance through the enhancement of proline biosynthesis.

## 4. Discussion

**Figure 9:**
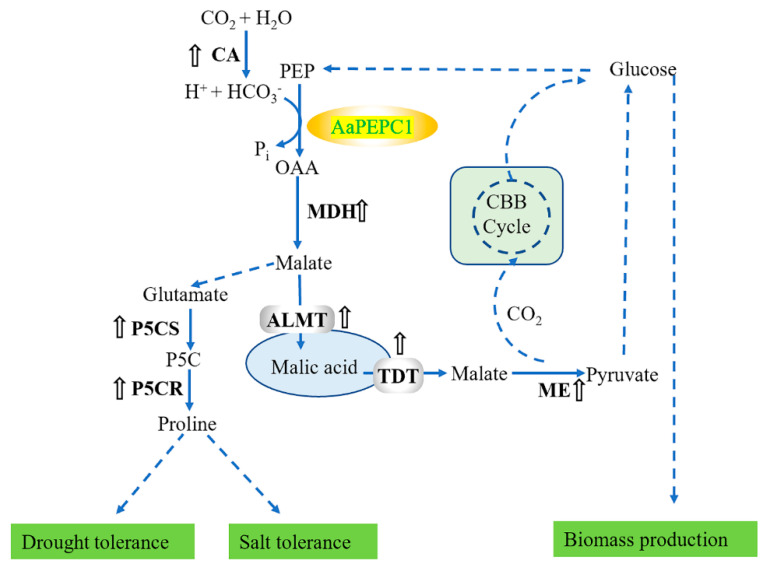
Regulation of photosynthetic CO_2_-fixation and abiotic stress tolerance in the ‘turbocharging’ plants with the *AaPEPC1*. Overexpression of the *AaPEPC1*, a highly abundant enzyme catalyzing the primary fixation of CO_2_ in CAM plants, increases photosynthetic CO_2_-fixation, and rewires the diel accumulation-depletion pattern of malate and glucose. The re-programed malate-dependent carboxylation leads to the feedback up-regulation of the orthologs of key CAM pathway genes, i.e., nocturnal carboxylation and diurnal decarboxylation modules. The increased malate content up-regulates pyrroline-5-carboxylate synthase (P5CS) and pyrroline-5-carboxylate reductase (P5CR), which results in higher proline accumulation. Proline accumulation enhances the salt and drought tolerance of the transgenic plants expressing *AaPEPC1*. Additionally, a higher glucose content produced from the photosynthetic source is transported as sucrose or glucose to sink tissues and organs to promote cell proliferation, elongation, and expansion, as well as to maintain energy and metabolic homeostasis, resulting in improved biomass production [64,65]. The up arrows indicate up-regulation of expression of genes coding these enzymes or content. CA: carbonic anhydrase; OAA: oxaloacetate; MDH: malate dehydrogenase; ALMT: aluminum-activated malate transporter; TDT: tonoplast dicarboxylate transporter; ME: malic enzyme; PEP: phospho*enol*pyruvate; and CBB: Calvin–Benson–Bassham.

The engineering of the water-conserving CO_2_-concentrating mechanism of CAM has been proposed as a potential strategy for improving photosynthetic CO_2_ fixation and abiotic stress tolerance in C_3_ plants [1,2,9]. In this study, we found that the overexpression of one single CAM gene *AaPEPC1* enhanced the performance of transgenic plants in multiple ways, including photosynthesis, biomass production, drought tolerance and salt tolerance, which yet not be reported. We determined the impacts of the ectopic expression of the *AaPEPC1* on C_3_-toward-CAM progression. Specifically, we identified a CAM-type *PEPC* (*AaPEPC1*) in *A. americana* and transformed the *AaPEPC1* gene into C_3_ plant tobacco. Compared with the WT and EV controls, the transgenic plants expressing *AaPEPC1* showed several interesting traits: (1) a higher malate content at the onset of the dark period, (2) higher leaf carbon isotope ratios (δ^13^C values), and (3) upregulated expression levels of the orthologs of several putative key CAM pathway genes (Figure 3 and Figure 4). These changes in photosynthetic carbon metabolism driven by *AaPEPC1* improved photosynthetic CO_2_-fixation, biomass production and tolerance to drought and salt stresses (Figure 9). These traits are not necessarily associated with CAM. Recently, Boxall, et al. [60] silenced *PEPC1* in a CAM plant species *Kalanchoë laxiflora* and found that the very low level of PEPC activity in the RNAi transgenic line was associated with the refixation of respiratory CO_2_ and malate accumulation. Daloso, et al. [61] reported that tobacco guard cells fix CO_2_ by both Rubisco and PEPC. The photosynthetic carbon metabolism changes in our study could be explained by PEPC-mediated CO_2_ fixation during the light period or the refixation of respiratory CO_2_, or an enhanced anaplerotic role for PEPC as in C_3_ plants. A CO_2_ labeled assay and metabolites analysis could be used to test this hypothesis and to identify the possible secondary products arising from *PEPC* overexpression in the future. Metabolite (e.g., glucose) sensing and signaling play important roles in regulating gene expression and controlling plant development [62,63]. We hypothesize that *AaPEPC1* overexpression perturbated the expression levels of metabolite signaling genes which subsequently caused the phenotypic changes (e.g., drought stress tolerance) in the transgenic tobacco plants. We found that the glucose, malate and proline contents were increased, and a few CAM-related genes were upregulated in the transgenic plants overexpressing *AaPEPC1*. Glucose, as a reducing sugar, is only ever present in modest concentrations, thus representing the partitioning of primary photosynthate or trafficking of reserves. A full starch–glucan–sucrose digestion could be performed to comprehensively evaluate the carbohydrate status in transgenic plants, and an integrative analysis of metabolomics, transcriptomics and proteomics could help to test this hypothesis in the future. This initial success provides a solid foundation for future effort to achieve a complete switch from C_3_ to CAM photosynthesis by engineering additional CAM-related genes involved in carboxylation, decarboxylation, stomatal movement, the glycolytic-gluconeogenic pathway and carbohydrate turnover modules [1,2,9].

It is interesting that *AaPEPC1* overexpression increased the transcript abundance of several other CAM-related genes, including *CA*, *MDH*, *ALMT*, *TDT* and *ME* (Figure 4). As a CO_2_-fixation enzyme, AaPEPC1 is not able to directly regulate the expression of other genes at the transcription level. We hypothesize that the change in cellular metabolic status caused by the *AaPEPC1* overexpression resulted in a rewiring of the regulatory network. This hypothesis can be tested through the transcriptomic and metabolomic analysis of gain-of-function and loss-of-function *AaPEPC1* mutants in the future. It was recently reported that phosphorylation of PEPC is essential for core circadian clock operation in the obligate CAM species *Kalanchoë fedtschenkoi* [20]. Therefore, it would be interesting to investigate the impact of *AaPEPC1* overexpression on the circadian rhythm in the transgenic plants in the future. In addition, on the premise of the coordinated regulation of multiple CAM-related genes by overexpressing one key gene such as *AaPEPC1*, we can argue that there is no need to transfer all the CAM pathway genes into C_3_ species; consequently, we just need to focus on the engineering of a small number of “master” genes like *AaPEPC1*, which can upregulate the expression of multiple other CAM-related genes.

Although PEPC is well-known as a key enzyme for CO_2_ fixation, its role in conferring resistance to salt stress in plants is not well defined [66]. In this study, we demonstrated that the overexpression of *AaPEPC1* significantly increased the salt tolerance in transgenic tobacco plants (Figure 6), likely a result of enhanced biosynthesis of proline (Figure 8), which plays important roles in regulating osmotic balance, activating ROS scavenging system, protecting membrane integrity and photosynthesis [11]. C_4_-type PEPC and C_3_-type PEPC have also been reported to be involved in drought tolerance improvement [67,68], but the mechanism is unclear. Thus far, the role of CAM-type PEPC in drought tolerance has not been reported yet. In this study, we found that overexpression of the *AaPEPC1* significantly increased the drought tolerance in the transgenic tobacco plants (Figure 7). Our results suggested that the improvement of photosynthetic carbon metabolism in the transgenic *AaPEPC1* plants enhanced proline metabolism pathway, which results in improved drought tolerance (Figure 9). In addition, compared to the previously published work for C_4_-type PEPC or C_3_-type PEPC overexpression in plants [67,68,69,70], this is the first time it has been shown that overexpression of one single *PEPC* gene enables the simultaneous improvement of photosynthesis and stress tolerance in plants.

Recently, photosynthesis and plant growth were significantly improved in tobacco plants by introducing a faster Rubisco of cyanobacterial origin [71], accelerating recovery from photoprotection [72], or engineering synthetic glycolate metabolism pathways [5]. However, none of these approaches enhanced tolerance to drought or salt stresses. In addition, previous genetic engineering efforts have reported progress in creating genetically-modified plants with enhanced tolerance to either drought stress [73,74] or salt stress [75,76,77], with limited success in conferring tolerance to both drought stress and salt stress in a single transgenic line. In this study, we created genetically modified tobacco plants that had enhanced performance in multiple aspects: photosynthesis, plant growth, drought tolerance, and salt tolerance. As such, we have provided new insights into the concept that the coordinated regulation of photosynthesis can increase biomass and stress tolerance [78]. These pleiotropic effects of *AaPEPC1*-overexpression open a new door to genetic improvement of crops for sustainable bioenergy and food production on marginal lands to alleviate the challenge caused by human population growth, urbanization, and global climate change.

In conclusion, we report the first successful effort of the engineering of a CAM pathway gene to improve photosynthetic CO_2_ fixation and abiotic stress tolerance in tobacco, the model C_3_ plant species. These findings have important implications for ultimate aspirations to engineer CAM into non-CAM crops as a means of improving productivity, and abiotic stress tolerance.

## Data Availability

Data presented in this study are contained in this article, or available upon request to the corresponding author.

## Supplementary Materials

The following are available online at https://www.mdpi.com/2073-4409/10/3/582/s1, Figure S1: Development of transgenic lines of tobacco overexpressing *Agave americana* PHOSPHO*ENOL*PYRUVATE CARBOXYLASE (AaPEPC1); Figure S2: Chlorophyll relative content in the leaves of transgenic plants expressing *Agave americana* PHOSPHO*ENOL*PYRUVATE CARBOXYLASE (*AaPEPC1*) (OE1, OE2) or empty vector (EV), and wild-types (WT) grown in pots under normal growth conditions. Table S1: Primers used in this study.

## Additional Information

This manuscript has been authored by UT-Battelle, LLC under Contract No. DE-AC05–00OR22725 with the U.S. Department of Energy. The United States Government retains and the publisher, by accepting the article for publication, acknowledges that the United States Government retains a non-exclusive, paid-up, irrevocable, world-wide license to publish or reproduce the published form of this manuscript, or allow others to do so, for United States Government purposes. The Department of Energy will provide public access to these results of federally sponsored research in accordance with the DOE Public Access Plan (http://energy.gov/downloads/doe-public-access-plan).
