# Supplementary material for: Overexpression of an Agave Phosphoenolpyruvate Carboxylase Improves Plant Growth and Stress Tolerance
Source: Cells. 2021 Mar 6;10(3):582. doi: 10.3390/cells10030582 (PMC7999111; doi:10.3390/cells10030582)
Supplement: Supplementary file 1 [file cells-10-00582-s001.pdf]

Supplementary Figures

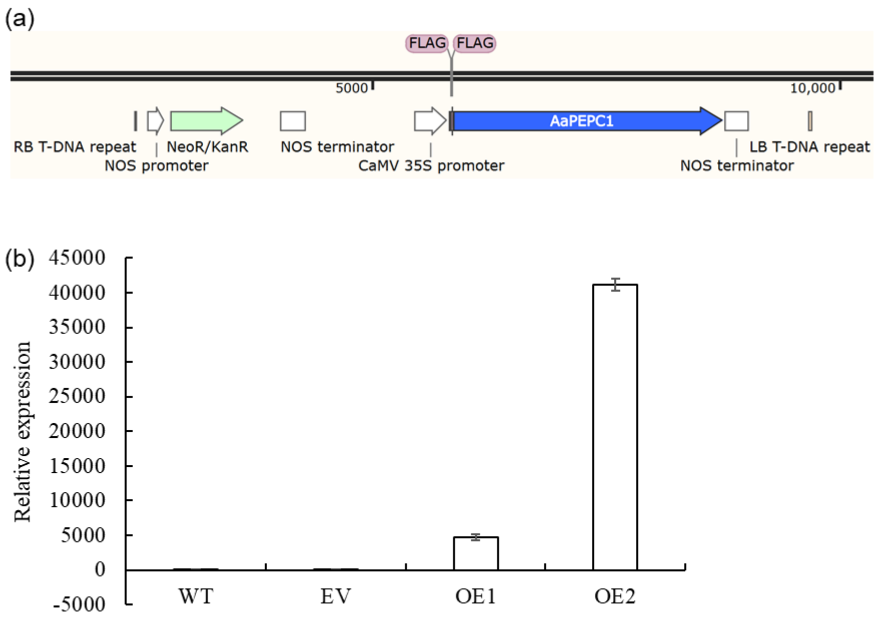

Supplementary Figure 1. Development of transgenic lines of tobacco overexpressing *Agave americana* PHOSPHO*ENOL*PYRUVATE CARBOXYLASE (*AaPEPC1*). (a) Schematic representation of the DNA construct developed for the overexpression of *AaPEPC1* in transgenic tobacco. CaMV35S: cauliflower mosaic virus 35S; NOS: nopaline synthase; KanR: kanamycin resistance gene as a selection marker for screening transgenic plants; “LB” and “RB”: the left and right borders of T-DNA, respectively. The image was generated with SnapGene software (http://www.snapgene.com/). **(b)** Quantitative reverse transcription PCR (qRT-PCR) analysis to detect the expression of *AaPEPC1* in transgenic plants expressing *AaPEPC1* (OE1, OE2), empty vector (EV), and wild-types (WT).

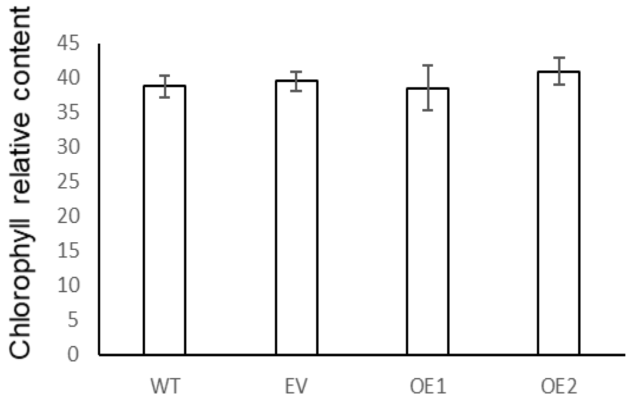

Supplementary Figure 2. Chlorophyll relative content in the leaves of transgenic plants expressing *Agave americana* PHOSPHO*ENOL*PYRUVATE CARBOXYLASE (*AaPEPC1*) (OE1, OE2) or empty vector (EV), and wild-types (WT) grown in pots under normal growth conditions. Values represent means ± SD.

Supplementary Tables

Table S1 Primers used in this study

Primer name	Primer sequence (5′-3′)
AaPEPC1_qPCR_F2	GCCTACAGGAGGAGGAGTACGCTG
AaPEPC1_qPCR_R2	CCTGACTGACTGAGTCGGATGTGC
NsyALMT_qRT_F1	AATGGTTCAGAGTATGGATTGG
NsyALMT_qRT_R1	TAAGTGTCGCACCTATGCTG
NsyCA4_qRT_F1	GTCAAAGCCCTAAGTTCTTGGT
NsyCA4_qRT_R1	ACCCACTCTTCAATGAAATCACTG
NsyMDH_qRT_F1	TTGGATATGCTCTTGTTCCGA
NsyMDH_qRT_R1	GCAACAACACCTTTGAGAAGAG
NsyME_qRT_F1	TGGCTTGTGGATTCAAAGGG
NsyME_qRT_R1	GGTTGGCTTAATGGTCTTAACAG
NsyP5CR_qRT_F1	TAATACTCAGGTGGTTGAAGACAG
NsyP5CR_qRT_R1	CAAAGTAAAGACAGTGGCGG
NsyP5CS_qRT_F1	CTTCAGGCACTTTCTTCCCA
NsyP5CS_qRT_R1	CATCAGCAACCTCCGTTCTC
NsyTDT_qRT_F1	TGGAACTGTTAGTGTCATGATGG
NsyTDT_qRT_R1	CTGGTGCAATGGCTAAGTATGG
Nsyl_Act97_qF1	TACCCAATTGAGCATGGAATTGTC
Nsyl_Act97_qR1	AACACAATACCGGTGGTACGA
